# Supplementary material for: The interactome of intact mitochondria by cross-linking mass spectrometry provides evidence for coexisting respiratory supercomplexes
Source: Mol Cell Proteomics. 2017 Dec 8;17(2):216–32. doi: 10.1074/mcp.RA117.000470 (PMC5795388; doi:10.1074/mcp.RA117.000470)
Supplement: Supplemental Data [file supp_RA117.000470_133922_0_supp_23978_fzffwf.zip › spectra_annotation/mito_spectra_annotation/169-1-1-1-107-1.pdf]

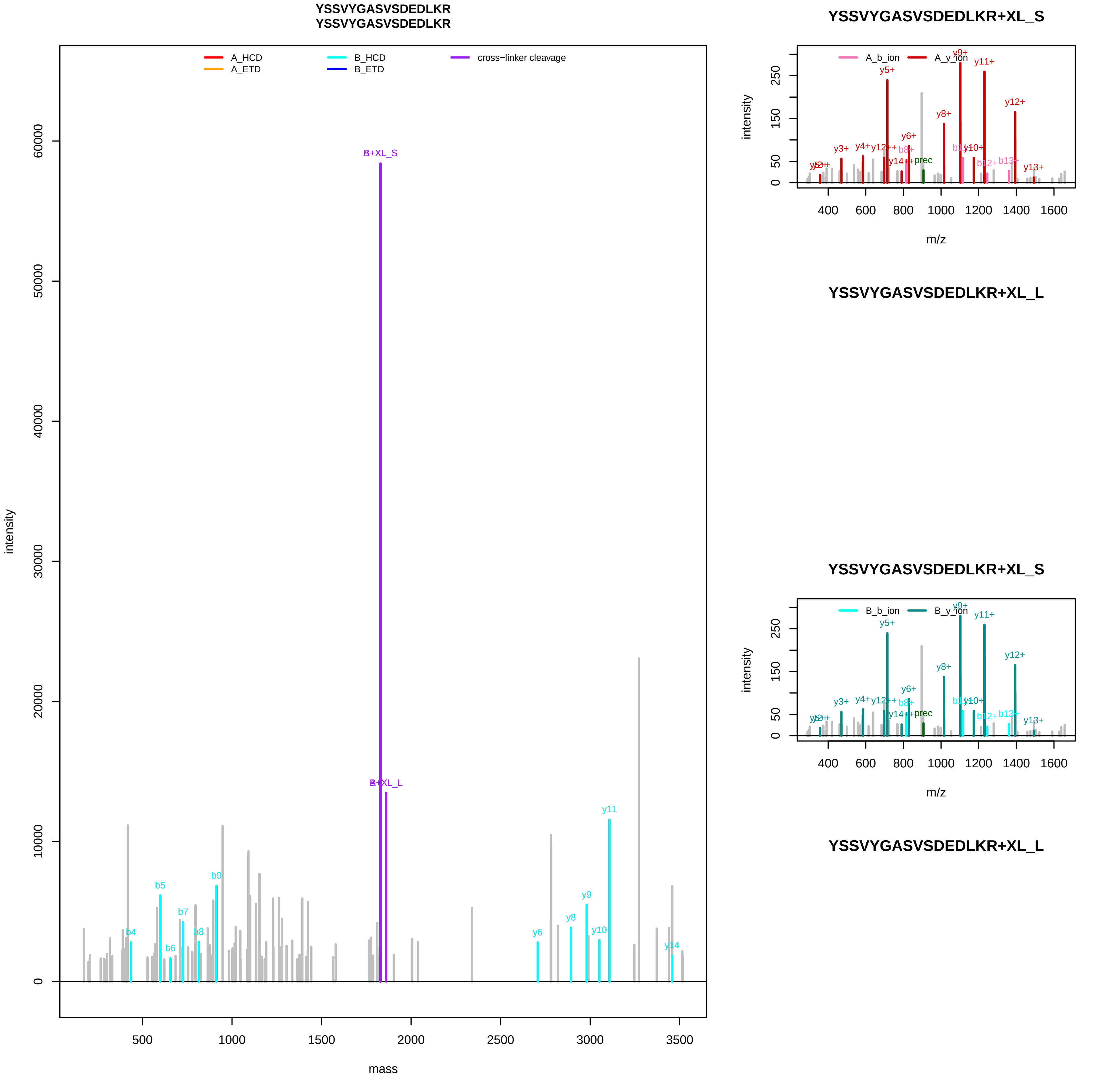
YSSVYGASVSDEDLKR
YSSVYGASVSDEDLKR
A_HCD
A_ETD
B_HCD
B_ETD
cross−linker cleavage
intensity
mass
B+XL_S
B+XL_L
b4
b5
b6
b7
b8
b9
y6
y8
y9
y10
y11
y14
YSSVYGASVSDEDLKR+XL_S
A_b_ion
A_y_ion
intensity
m/z
y5+
y9+
y11+
y8+
y12+
y3+
y4+
y12++
y6+
b8+
y14++
prec
b11+
y10+
b12+
b13+
y13+
YSSVYGASVSDEDLKR+XL_L
YSSVYGASVSDEDLKR+XL_S
B_b_ion
B_y_ion
intensity
m/z
YSSVYGASVSDEDLKR+XL_L
